# Editorial for the Special Issue “Breast Cancer—Therapeutic Challenges, Research Strategies and Novel Diagnostics”

**DOI:** 10.3390/cancers15184611

**Published:** 2023-09-18

**Authors:** Naiba Nabieva

**Affiliations:** 1 Department of Gynecology and Obstetrics, Friedrich-Alexander-Universität Erlangen-Nürnberg, 91054 Erlangen, Germany; naiba.nabieva@fau.de; 2 GynPraxis, 91054 Erlangen, Germany

Worldwide, breast cancer affects over 2 million women a year, with a rising burden [1]. Thanks to the breast cancer research of recent decades, effective methods have been established, allowing the mortality rates of patients with this disease to decrease more and more. The aim of this Special Issue was to gather original articles and reviews demonstrating therapeutic challenges, research strategies and novel diagnostics in breast cancer.

In a retrospective multicenter registry, the Turkish Oncology Group evaluated time-related differences in treatment patterns and outcome in a real-world patient population with metastatic breast cancer (mBC) over a ten-year timeframe. Due to the incorporation of novel agents, the HER2+ subgroup showed a significant survival benefit, while triple-negative mBC (TNBC) patients still have the worst prognosis [2].

Gong et al. analyzed the impact of temporal and spatial tumor heterogeneity assessed using the discordance between primary and metastatic immunohistochemistry results and the 18F-FDG uptake on PET/CT, respectively, on the treatment outcome of patients with HER2+ mBC treated with pyrotinib. The results showed that temporal and spatial HER2 heterogeneity were predictive of poorer outcomes of pyrotinib treatment [3]. Xie et al. found that the novel 18F-FES PET/CT method could also identify mBC patients with heterogeneity in estrogen receptor expression. In these patients, chemotherapy showed a better efficacy compared with endocrine treatment [4]. However, the best method to evaluate tumor heterogeneity in clinical practice still needs to be identified.

Since TNBC shows the worst prognosis and limited treatment options, exploring novel molecular targets is urgently needed. Li et al. demonstrated that the novel oncogene LEM Domain Containing 1 (LEMD1) is highly expressed in TNBC and could act as a therapeutic target as its knockdown renders TNBC cells more sensitive to paclitaxel [5]. Also, Pannexin 1 (PANX1) has been found to be a poor prognostic factor in breast cancer; however, its role remains unknown. Chen et al. could show that PANX1 had high expression in basal-like breast cancer, and this in turn is associated with high tumor-associated neutrophil infiltration and adenosine production to induce local immunosuppression in tumor microenvironment [6].

Furthermore, it is interesting to learn more about the worldwide situation on *BRCA1/2* germline mutation testing. According to Mahtani et al., real-world data from the United States, Europe and Israel reveal that 73%, 42% and 99% of HER2− advanced breast cancer (aBC) patients were tested for *BRCA1/2*, respectively. In the US and Europe, patients who were not tested versus those who were tested were older, more likely to have HR+/HER2− aBC than TNBC and less likely to have a known family history of *BRCA1/2*-related cancer. Efforts should be made to improve *BRCA1/2* testing rates in affected countries [7].

In early breast cancer (eBC), advancements in diagnostic and localization methods are of special interest. Early detection of breast cancer in asymptomatic women through screening is an important strategy in reducing its burden. The systematic review by Velentzis et al. assessed, using a variety of methods, how accurately breast cancer risk assessment tools can group women eligible for screening within a population, into risk groups, so that each group could potentially be offered a screening protocol with more benefits and less harm compared to current age-based screening [8]. Nicosia et al. compared the diagnostic performance of Contrast-Enhanced Mammography (CEM) versus Digital Mammography (DM), and of CEM versus DM + Digital Breast Tomosynthesis (DBT), performed in the same group of patients over the same period of time in a screening setting. CEM offered a lower average glandular dose than DM protocols with added tomosynthesis. Its diagnostic performance was no less than that of DM + DBT letting the use of CEM appear promising in screening settings in dense breasts and high-risk patients [9]. Furthermore, artificial intelligence will play an important role in the detection of lesions. Vrdoljak et al. trained and evaluated several machine-learning models with the aim of predicting breast cancer lymph node metastases in patients eligible for neoadjuvant treatment. According to the authors, the models achieved a good performance in assessing the lymph node status so that such an approach could lead to more accurate disease stage prediction and consecutively better treatment selection, especially for NST patients where radiological and clinical findings are often the only method of lymph node assessment [10]. Regarding localization methods, the review of Banys-Paluchowski et al. provides an overview of current localization techniques for non-palpable breast lesions, associated knowledge gaps and potential methods to close these [11].

When it comes to HR+ breast cancer, CDK4/6 inhibitors are the first substances in almost two decades to substantially change the standard of care not only for aBC patients, but also for those with an early disease stage. In their review, Nabieva et al. discuss the recent history, current role, future directions and opportunities of this substance class [12]. However, despite advancements in endocrine treatment, especially in HR+ eBC patients often the question arises of whether treatment escalation in terms of a chemotherapy is necessary. Dannehl et al. assessed whether the multigene-expression assay Oncotype DX^®^ that has been validated in two large clinical phase III trials, effectively reduces treatment escalation in a real-world setting. The authors could demonstrate that, using Oncotype DX^®^, absolute adjuvant chemotherapy recommendation can be reduced by nearly 15% [13].

And while chemically produced drugs are the standard of care, Chavda et al. emphasize in their review the anticancer activity of phytochemical-instigated and phytochemical-loaded nanocarriers against breast cancer both in vitro and in vivo. The authors discuss the selective targeted delivery of phytofabricated nanocarriers to cancer cells and consider research gaps, recent developments and the drugability of phytoceuticals [14].

Having spoken intensively about the therapy of breast cancer patients, it has to be mentioned that a well-treated patient is not automatically a healthy one. A lot depends also on cognitive and psychological well-being. Having undergone the pandemic and living in a world becoming more and more digitalized, telemedicine approaches are gaining more interest. Giustiniani et al. conducted a systematic review to clarify the effectiveness of telerehabilitation for treating the cognitive and psychological difficulties of breast cancer patients [15].

In conclusion, the collection of articles in the Special Issue “Breast Cancer—Therapeutic Challenges, Research Strategies and Novel Diagnostics” has made substantial contributions to our comprehension of breast cancer. The authors shed light on known as well as emerging diagnostic and therapeutic approaches, and various other aspects associated with this global disease burden. I hope that healthcare professionals and researchers working in this field will find it helpful.

## References

[1] Heer E., Harper A., Escandor N., Sung H., McCormack V., Fidler-Benaoudia M.M. (2020). Global burden and trends in premenopausal and postmenopausal breast cancer: A population-based study. Lancet Glob. Health.

[2] Dogan I., Aksoy S., Cakar B., Basaran G., Ercelep O., Molinas Mandel N., Korkmaz T., Gokmen E., Sener C., Aydiner A. (2023). Demographic and Clinical Features of Patients with Metastatic Breast Cancer: A Retrospective Multicenter Registry Study of the Turkish Oncology Group. Cancers.

[3] Gong C., Liu C., Tao Z., Zhang J., Wang L., Cao J., Zhao Y., Xie Y., Hu X., Yang Z. (2022). Temporal Heterogeneity of HER2 Expression and Spatial Heterogeneity of 18F-FDG Uptake Predicts Treatment Outcome of Pyrotinib in Patients with HER2−Positive Metastatic Breast Cancer. Cancers.

[4] Xie Y., Du X., Zhao Y., Gong C., Hu S., You S., Song S., Hu X., Yang Z., Wang B. (2022). Chemotherapy Shows a Better Efficacy Than Endocrine Therapy in Metastatic Breast Cancer Patients with a Heterogeneous Estrogen Receptor Expression Assessed by 18F-FES PET. Cancers.

[5] Li X., Jiang S., Jiang T., Sun X., Guan Y., Fan S., Cheng Y. (2023). LEM Domain Containing 1 Acts as a Novel Oncogene and Therapeutic Target for Triple-Negative Breast Cancer. Cancers.

[6] Chen W., Li B., Jia F., Li J., Huang H., Ni C., Xia W. (2022). High PANX1 Expression Leads to Neutrophil Recruitment and the Formation of a High Adenosine Immunosuppressive Tumor Microenvironment in Basal-like Breast Cancer. Cancers.

[7] Mahtani R., Niyazov A., Arondekar B., Lewis K., Rider A., Massey L., Lux M.P. (2022). BRCA1/2 Mutation Testing in Patients with HER2−Negative Advanced Breast Cancer: Real-World Data from the United States, Europe, and Israel. Cancers.

[8] Velentzis L.S., Freeman V., Campbell D., Hughes S., Luo Q., Steinberg J., Egger S., Mann G.B., Nickson C. (2023). Breast Cancer Risk Assessment Tools for Stratifying Women into Risk Groups: A Systematic Review. Cancers.

[9] Nicosia L., Bozzini A.C., Pesapane F., Rotili A., Marinucci I., Signorelli G., Frassoni S., Bagnardi V., Origgi D., De Marco P. (2023). Breast Digital Tomosynthesis versus Contrast-Enhanced Mammography: Comparison of Diagnostic Application and Radiation Dose in a Screening Setting. Cancers.

[10] Vrdoljak J., Boban Z., Barić D., Šegvić D., Kumrić M., Avirović M., Perić Balja M., Periša M.M., Tomasović Č., Tomić S. (2023). Applying Explainable Machine Learning Models for Detection of Breast Cancer Lymph Node Metastasis in Patients Eligible for Neoadjuvant Treatment. Cancers.

[11] Banys-Paluchowski M., Kühn T., Masannat Y., Rubio I., de Boniface J., Ditsch N., Karadeniz Cakmak G., Karakatsanis A., Dave R., Hahn M. (2023). Localization Techniques for Non-Palpable Breast Lesions: Current Status, Knowledge Gaps, and Rationale for the MELODY Study (EUBREAST-4/iBRA-NET, NCT 05559411). Cancers.

[12] Nabieva N., Fasching P.A. (2023). CDK4/6 Inhibitors—Overcoming Endocrine Resistance Is the Standard in Patients with Hormone Receptor-Positive Breast Cancer. Cancers.

[13] Dannehl D., Engler T., Volmer L.L., Staebler A., Fischer A.K., Weiss M., Hahn M., Walter C.B., Grischke E.-M., Fend F. (2022). Recurrence Score&reg; Result Impacts Treatment Decisions in Hormone Receptor-Positive, HER2−Negative Patients with Early Breast Cancer in a Real-World Setting&mdash;Results of the IRMA Trial. Cancers.

[14] Chavda V.P., Nalla L.V., Balar P., Bezbaruah R., Apostolopoulos V., Singla R.K., Khadela A., Vora L., Uversky V.N. (2023). Advanced Phytochemical-Based Nanocarrier Systems for the Treatment of Breast Cancer. Cancers.

[15] Giustiniani A., Danesin L., Pezzetta R., Masina F., Oliva G., Arcara G., Burgio F., Conte P. (2023). Use of Telemedicine to Improve Cognitive Functions and Psychological Well-Being in Patients with Breast Cancer: A Systematic Review of the Current Literature. Cancers.

